# Clinical assessment of dental color during dehydration and rehydration by various dental photography techniques

**DOI:** 10.1007/s10266-025-01081-w

**Published:** 2025-03-10

**Authors:** Cigdem Haciali, Bora Korkut, Funda Yanikoglu

**Affiliations:** 1 Department of Restorative Dentistry, Faculty of Dentistry, Marmara University, Istanbul, Türkiye; 2 Department of Restorative Dentistry, Faculty of Dentistry, Istanbul Kent University, Istanbul, Türkiye

**Keywords:** Photography, Dehydration, Rehydration, Tooth color, Cross-polarization, White balance

## Abstract

Dental color selection is an important clinical challenge in restorative dentistry due to the alterations related to dehydration and rehydration. This clinical study aimed to quantitatively monitor the color changes between different levels of tooth dehydration and rehydration using different dental photography techniques. Color assessments of the right central incisor were performed using a hybrid-type clinical spectrophotometer (RayPlicker; RP) as control, a dental photography kit (DP), and a mobile dental photography kit (MDP). Cross-polarization filter (CP) and white balance card (WB) were also used in combinations with DP and MDP. Therefore, the effectiveness of eight different photography techniques (DP, DP-WB, DP-CP, DP-WB-CP, MDP, MDP-WB, MDP-CP, and MDP-WB-CP) were investigated. The color measurements during dehydration (at initial, 1, 2, 3, 5, and 15 min) and rehydration (at 30, 60 min, and 24 h) were performed with all techniques. Color changes (∆E_00_*) were calculated according to the CIEDE2000 formula by comparing the outcomes with the initial values. Regarding the dehydration values by RP, the color changes until the 3rd min were imperceptible (∆E < 0.8) and significantly lower than the 5th min (*p* < 0.001). The changes were perceptible but acceptable at the 5th min, while it was not even acceptable (∆E ≥ 1.8) at the 15th min. Regarding the rehydration values by RP, the color changes were perceptible and significantly higher at the 30th and 60th min than at the 24th h (*p* < 0.001). The color changes for 70% of the teeth were below 0.8 at the 24th h. Very high agreements were observed between the assessments by DP/CP, DP/WB/CP, MDP/CP, and MDP/WB/CP techniques, and the RP (*p* < 0.001 for each assessment method). The clinical color change was considered imperceptible until 3 min of dehydration. However, it became perceptible after 5 min of dehydration and not clinically acceptable after 15 min. Following 15 min of dehydration, the natural tooth color may not be completely reversed even after rehydrating for 24 h. CP filters were considered essential tools when using dental photography or mobile dental photography devices for dental color assessment.

## Introduction

Dental color selection can be performed subjectively by visual inspection or objectively by digital shade-taking devices in clinical dental practice [1]. Visual inspection depends on the interaction between light and the dental structure thereby on the clinician’s subjective decision [2]. The most common technique is digital shade-taking devices such as a spectrophotometer, a colorimeter, or, more recently, a digital dental camera kit [3]. These were considered to perform a more accurate, and reproducible objective color analysis clinically [4, 5]. Professional and mobile dental photography devices were also used together with the cross-polarization filters and/or white balance calibration cards to analyze the clinical dental color quantitatively [1, 2, 4–7]. The cross-polarization filters were considered effective tools to eliminate unwanted light reflections and provide more standardized and homogeneous lighting on the tooth surface with decreased white opacities for more detailed and objective analyses [1, 2, 6–9].

The CIE L*a*b* color space developed by CIE (Commission Internationale de Leclaire—CIE) is a common system to quantitatively analyze dental color [1]. Whereas, to achieve a better correlation with visual perception, recently ISO (International Standard Organization) and CIE have both recommended the use of CIEDE 2000 color space [4]. The ∆E* is the color difference, or the distance separating two points of color, and recently the clinical perceptible threshold (PT) for dental color was considered ≥ 0.8, and the clinically acceptable threshold (AT) was ≥ 1.8 [10].

Dehydration causes alterations in dental color [11, 12]. Natural dental tissues inevitably get dehydrated during various dental procedures, such as restorative field isolation techniques [13, 14]. Especially, rubber-dam isolation was considered the most effective factor for dehydration due to the color change above the acceptable threshold (AT ≥ 1.8) [11–17]. Alamé et al. [16] observed significant color changes above the AT at the 10th, 20th, and 30th min of rubber-dam isolation. Burki et al. [14] and Ibrahim and Abou Steit [17] reported similar outcomes for the 10th and 30th min and Russel et al. [11] for the 15th min of dehydration under the rubber-dam isolation. The change is mainly due to the filling of the interprismatic spaces with air instead of saliva, which alters the refractive index of the tissue [12, 15]. The increase in enamel opacity masks the underlying dentin color and makes the tooth seem whiter in color [11]. Although the change is reversible, it may adversely affect the color perception of the clinician, which may lead to a false assessment of tooth color during the restorative treatment [18]. The success in dental color matching is impacted by factors such as clinician’s viewing conditions, eye fatigue, age, clinical experience, and the presence or absence of color vision deficiencies [12]. Even dental prophylaxis was reported to affect the dental color assessment regarding both visual and instrumental methods [19]. Therefore, precise color selection while avoiding dehydration is a crucial procedure in dental practice to achieve high-end esthetic results [15].

There is a lack of information in the literature regarding the very short-term clinical effect of dehydration and the reverse effect of rehydration quantitatively [11, 12, 14]. Besides there are only a limited number of clinical studies in the literature reporting the efficiency of different dental photography equipment on quantitative tooth color selection [20, 21]. Therefore, this clinical study aimed to quantitatively monitor the color changes of maxillary central incisors after various dehydration and rehydration periods using different dental photography techniques. The hypotheses of the study were set as follows: (1) the clinical perception of tooth color is not affected by the level of tooth dehydration; (2) the clinical perception of tooth color is not affected by the level of tooth rehydration; (3) there is no significant difference in clinical color detection between dental photography and spectrophotometer techniques.

## Materials and methods

This clinical study was approved by the local ethics committee (Date: 23.12.2021 and protocol number: 2021/32) and registered in the U.S. National Library of Medicine (ClinicalTrials.gov Identifier: NCT05620277). It was designed and conducted in a university clinic between January and December of 2022. Power analysis according to the G*Power 3.1.9.7 software program indicated that a minimum sample size of 28 patients was necessary for the study for an effect size of *f* = 0.376 at a confidence level 1-α = 0.95 and desired power 1-β = 0.95 [13, 22]. Male or female adult participants at the ages of 20–40 were included in the study among the university’s active clinical staff. The participants who had systemic disease, advanced periodontal problems, partial and full prosthetic dentures, existing direct or indirect restorations, caries lesions, or palatal retainers, internal discolorations such as fluorosis and tetracycline discoloration, white lesions such as hypomineralization and initial caries lesions on the maxillary incisors were excluded from the study. The smokers and the ones who recently received vital/devital tooth bleaching procedures were also excluded from the study. Finally, a total of 30 participants were included in the study. All the selected participants were informed that their records would be taken non-invasively using various dental color measurement techniques at different periods in the clinic and they would be used for the investigation. Accordingly, each participant signed an informed consent form. One week before the color assessments, every participant was submitted to professional dental prophylaxis using an ultrasonic scaler and a nylon brush with prophylaxis paste (Cleanic, Kerr Dental, USA).

### Color assessment equipment and clinical application protocols

**Fig. 1:**
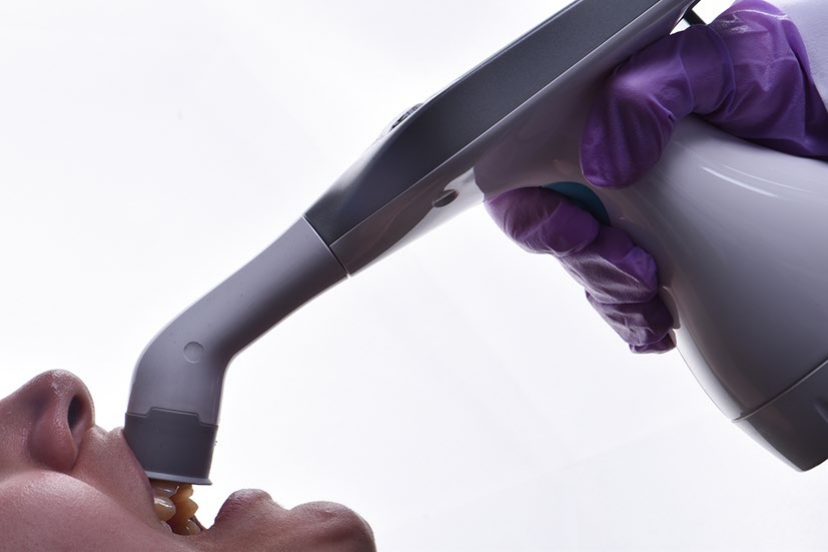
Clinical dental color measurements with RayPlicker (Borea, France)

**Fig. 2:**
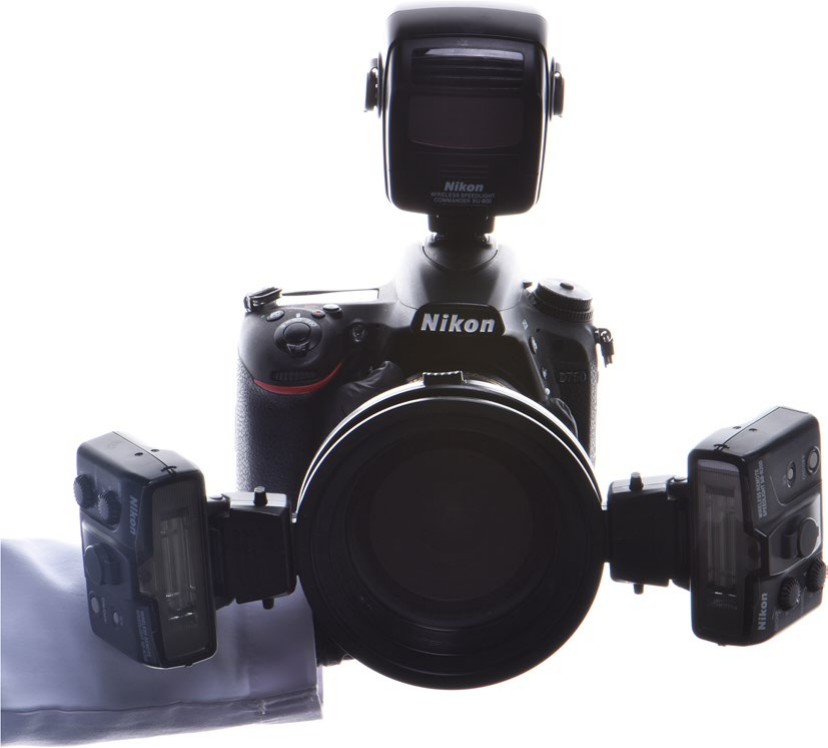
Digital dental photography kit (D750 body, R1C1 Speedlight SB-R200 flash, 105 mm AF-S Micro Nikkor VR lens, Nikon, Japan)

**Fig. 3:**
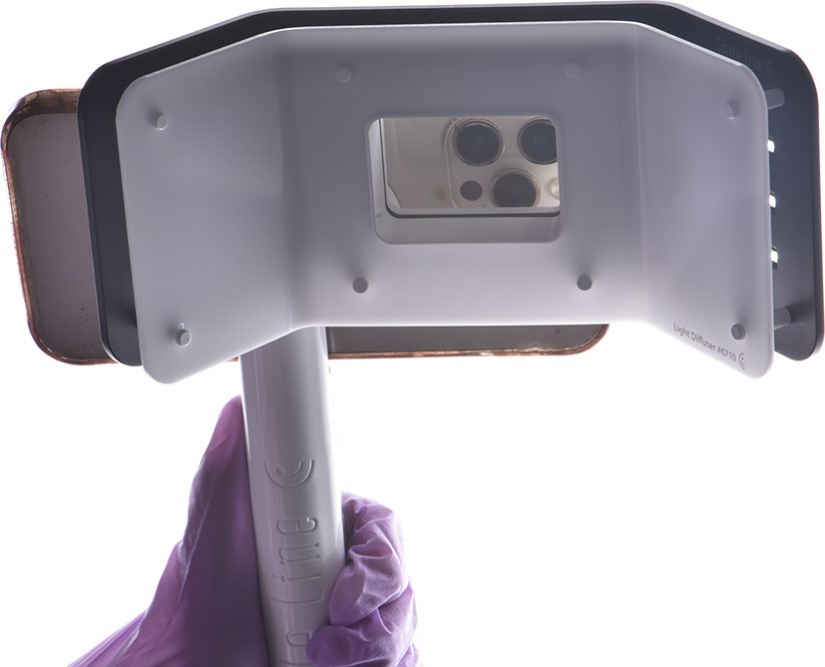
Mobile dental photography kit including MDP II device (Smile Line, Switzerland) and (iPhone 15 Pro Max, Apple, USA)

**Fig. 4:**
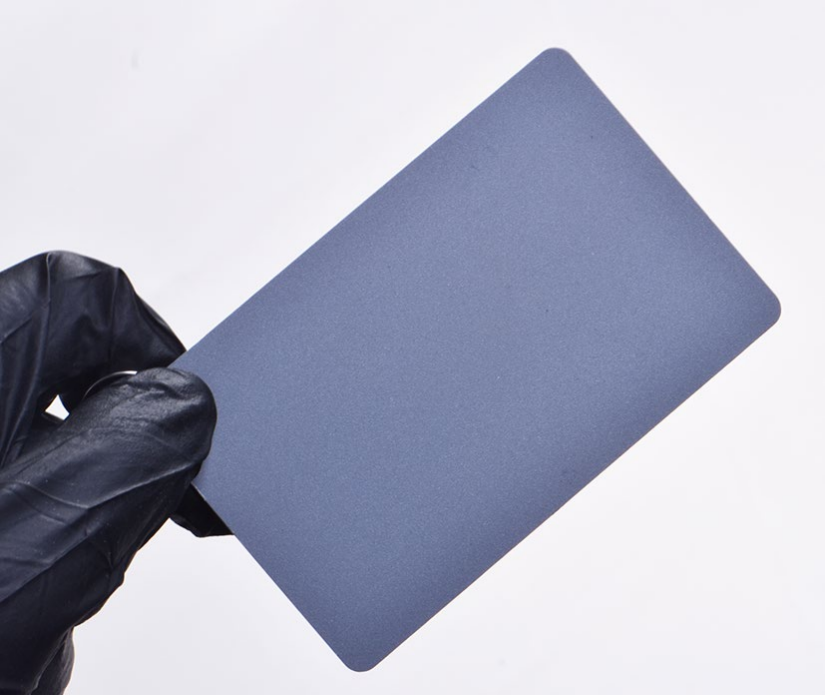
White balance calibration card (E-Lab, Emulation, Germany)

**Fig. 5:**
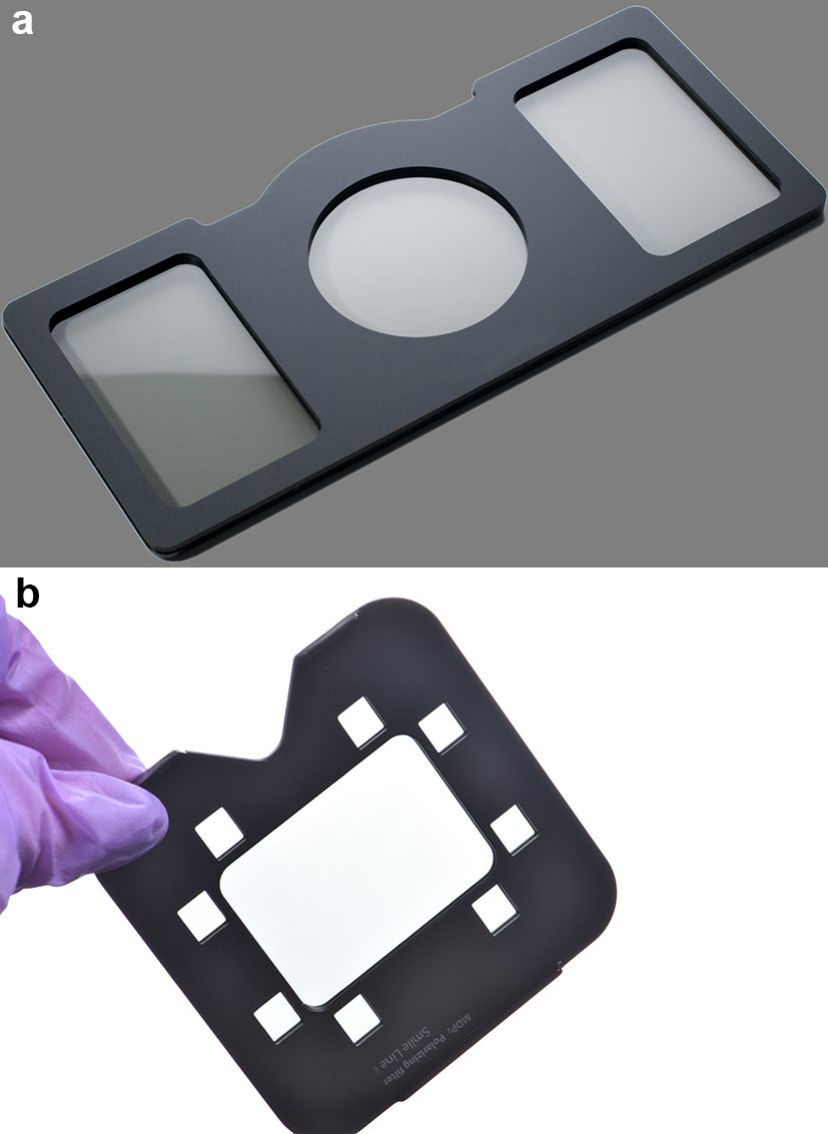
**a** Polar frame (Dens-Natura, Australia) CP filter, **b** CP filter of the MDP II device

**Fig. 6:**
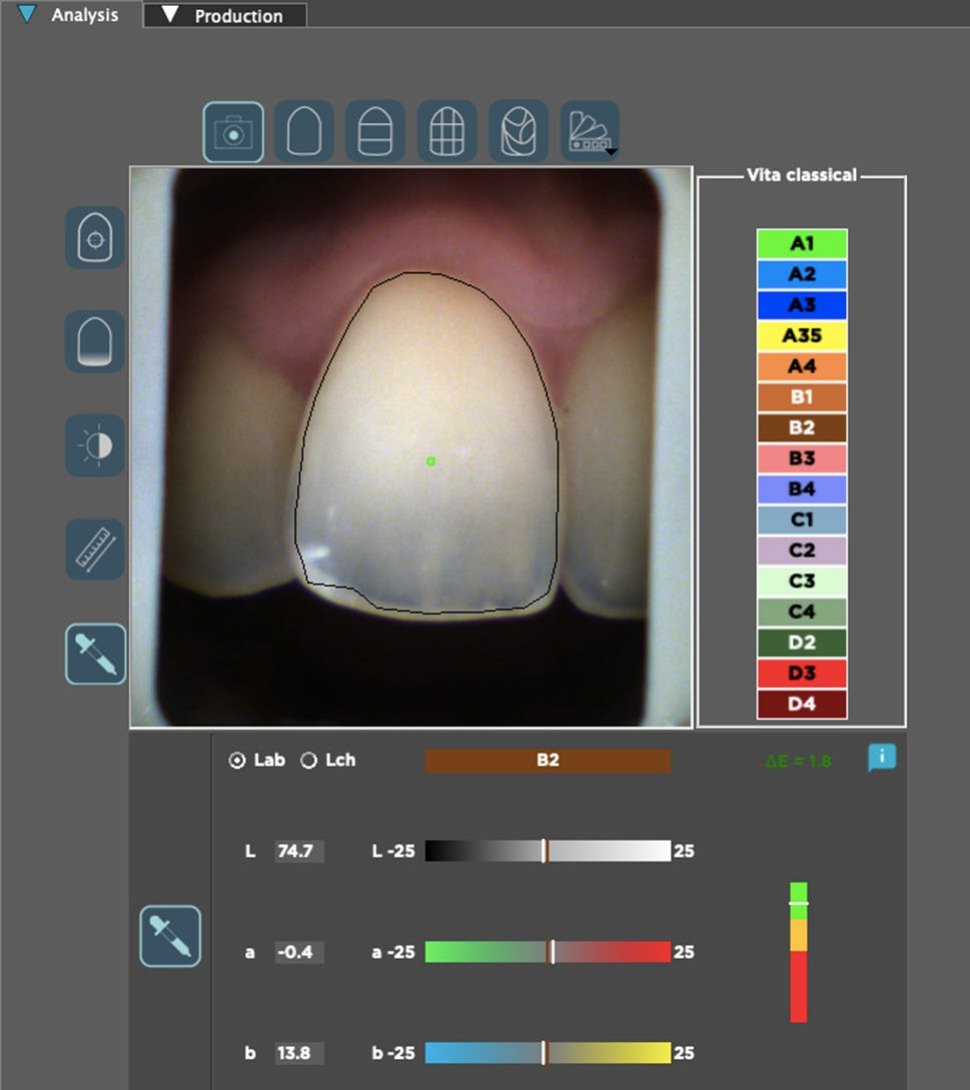
Quantitative color assessment with the RP software

The dental photography equipment used in the study is presented in Table 1. A hybrid-type clinical spectrophotometer (RayPlicker, Borea, France; RP) was used as a control in this study (Fig. 1). A dental photography kit (D750 body, AF-S Micro Nikkor VR 105 mm lens, R1C1 Speedlight SB-R200 flash, Nikon, Japan; DP), and a mobile dental photography kit (Smile Lite MDP II, Smile Line, Switzerland and iPhone 15 Pro Max, Apple, USA; MDP) were the investigated dental photography devices (Figs. 2 and 3). The DP and MDP were used in combination with a gray reference card (E-Lab, Emulation, Germany; WB) and compatible cross-polarization filters (Polar Frame, Dens-Natura, Australia, and MDP cross-polarization filter, CP) (Figs. 4, 5, and 6). The reflector light of the dental unit (Compact i Classic, Planmeca, Finland) was not used to ensure standardization of illumination during the shooting and the measurements. In addition, the curtains of the clinic were closed, and the ambient light sources (EF-200 V LED, Jinbei, China) remained constant at 5500 Kº, with the Color Rendering Index [CRI] of 95 [18]. The dental unit was reclined almost horizontally to the relative ground at a 60 cm distance from the floor. The adjusted position was saved in one of the inclination modes (Mode D) of the dental unit, and the headrest position was also fixed. Thereby, the same adjustments were used throughout the entire study to ensure the standardization of the photographs. Therefore, the participants were seated in the same fixed dental unit position [3].

**Table 1:** Dental photography equipment for the present study

Equipment	Brand/place of production	Code
Clinical spectrophotometer	RayPlicker, Borea, Limoges, France	RP
Dental photography kit	DSLR D750 (full-frame); 105 mm AF-S Micro Nikkor VR lens; R1C1 SB-R200 Speedlight dual flash; Nikon, Tokyo, Japan	DP
Mobile dental photography kit	Smart Lite MDP II, Smile Line, Switzerland; iPhone 15 Pro Max, Apple Inc., USA	MDP
Cross-polarization filter	Polar Frame, Dens-Natura, Australia	CP
White balance calibration card	Gray card, E-Lab, Emulation, Freiburg, Germany	WB

The dental color assessment techniques and clinical application protocols are presented in Table 2. Eight different dental photography techniques (DP, DP/WB, DP/CP, DP/WB/CP, MDP, MDP/WB, MDP/CP, and MDP/WB/CP) were investigated other than the control (RP). The participants brushed their teeth for 2 min without toothpaste before the measurements. All the photographs were taken by a single restorative dentistry instructor with 5 years of clinical experience at twelve o’clock position and 70–80 cm above the patient’s head (the distance between the camera objective and the tooth’ labial surface was 70–80 cm depending on the size of the patient’s head), regardless of the dental photography technique [23]. The instructor always stayed straight during the photo shoot to ensure standardization in the distance [3]. A limitation of photography standardization was the positioning of the patient’s head. However, it was minimized by checking the previous photography of the patient on a computer screen while shooting the new one.

**Table 2:** Color measurement techniques and clinical application protocols

Color assessment technique	Clinical application	Dehydration assessment period (s/min)	Rehydration assessment period (min/h)
RP	Dental unit is parallel to the ground. Device is positioned labially on maxillary right central incisor, contacting the labial surface and cervical gingiva	15 s, 1 min, 2 min, 3 min, 5 min, 15 min	30 min, 60 min and 24 h
DP	A full-frame DSLR camera and 105 mm macro-lens are used with dual flashes that are inclined at 45 degree. Shutter speed is set at 1/125, aperture at f25, and ISO at 200. An intraoral photograph is taken from the 12 o’clock position, at 70–80 cm distance to the maxillary right central incisor’s labial surface	15 s, 1 min, 2 min, 3 min, 5 min, 15 min	30 min, 60 min and 24 h
DP + WB	A full-frame DSLR camera, 105 mm macro-lens, and dual flashes at 45 degree are used. Shutter speed is set at 1/125, aperture at f25, and ISO at 200. An intraoral photograph is taken from the 12 o’clock position, at 70–80 cm distance to the maxillary right central incisor’s labial surface while holding a gray reference card close to the incisal edge of the tooth	15 s, 1 min, 2 min, 3 min, 5 min, 15 min	30 min, 60 min and 24 h
DP + CP	A full-frame DSLR camera, 105 mm macro-lens, dual flashes at 45 degree, and cross-polarization filter is used. Shutter speed is set at 1/125, aperture at f25, and ISO at 200. An intraoral photograph is taken from the 12 o’clock position, at 70–80 cm distance to the maxillary right central incisor’s labial surface	15 s, 1 min, 2 min, 3 min, 5 min, 15 min	30 min, 60 min and 24 h
DP + WB + CP	A full-frame DSLR camera, 105 mm macro-lens, dual flashes at 45 degree, and cross-polarization filter is used. Shutter speed is set at 1/125, aperture at f25, and ISO at 200. An intraoral photograph is taken from the 12 o’clock position, at 70–80 cm distance to the maxillary right central incisor’s labial surface while holding a gray reference card close to the incisal edge of the tooth	15 s, 1 min, 2 min, 3 min, 5 min, 15 min	30 min, 60 min and 24 h
MDP	Smile Lite MDP II device is used with an iPhone 15 Pro Max. Twin flashes of the MDP II and 2X optical zoom of the iPhone are used. An intraoral photograph is taken from the 12 o’clock position, at 70–80 cm distance to the maxillary right central incisor’s labial surface	15 s, 1 min, 2 min, 3 min, 5 min, 15 min	30 min, 60 min and 24 h
MDP + WB	Smile Lite MDP II device is used with an iPhone 15 Pro Max. Twin flashes of the MDP II and 2X optical zoom of the iPhone are used. An intraoral photograph is taken from the 12 o’clock position, at 70–80 cm distance to the maxillary right central incisor’s labial surface while holding a gray reference card close to the incisal edge of the tooth	15 s, 1 min, 2 min, 3 min, 5 min, 15 min	30 min, 60 min and 24 h
MDP + CP	Smile Lite MDP II device is used with an iPhone 15 Pro Max. Middle single flash and cross-polarization filter of the MDP II and 2X optical zoom of the iPhone are used. An intraoral photograph is taken from the 12 o’clock position, at 70–80 cm distance to the maxillary right central incisor’s labial surface	15 s, 1 min, 2 min, 3 min, 5 min, 15 min	30 min, 60 min and 24 h
MDP + WB + CP	Smile Lite MDP II device is used with an iPhone 15 Pro Max. Twin flashes and cross-polarization filters of the MDP II and 2X optical zoom of the iPhone are used. An intraoral photograph is taken from the 12 o’clock position, at 80 cm distance to the maxillary right central incisor’s labial surface while holding a gray reference card close to the incisal edge of the tooth	15 s, 1 min, 2 min, 3 min, 5 min, 15 min	30 min, 60 min and 24 h

Only 5 participants were assessed in a day. In addition, only one assessment technique was used per participant in a day to avoid the accumulated dehydration effect. Thus, the participants were called to the clinic again on another day to perform the rest of the color assessment techniques. Regarding the dehydration photographs, the lips of the participants were retracted by clear lips and cotton rolls. A stopwatch was used to arrange the exact color measurement periods. The retraction procedure was completed within 15 s, and immediately after that, the initial assessment was performed depending on the selected technique. Following that, the records for the 1st min, 2nd min, 3rd min, 5th min, and 15th min were collected. Then, the retractors and the cotton rolls were removed, the participant closed the mouth, and the teeth were rehydrated by the saliva naturally. The rehydration assessments were performed using the same technique 30 and 60 min after the removal. In addition, the rehydration assessments at 24 h were performed on the following day at the exact hour for each participant and each color assessment technique.

RP is a contact-type clinical color measurement device that can quantitatively analyze dental color using both spectrophotometry and CP photography features simultaneously [24]. RP has similar features to the predecessor SpectroShade device which was considered the gold standard in the literature [25, 26]. The soft gray tip of the RP device was placed in contact with the middle third labial surface of the maxillary right central incisors, including the cervical gingiva and then the image was captured. The settings for DP were fixed at shutter speed (1/125), aperture (f25), and ISO 200 for standardization. The dual flashes were inclined inside at 45 degrees and fixed. During the MDP photography, the smartphone’s optical zoom was fixed at 2X for the shooting [7]. The smartphone camera settings were fixed at f/16 with no use of an integrated flashlight [8]. The gray reference card (WB) was used to adjust the white balance of the image temperature. Accordingly, the assistant held the card during the photography shooting with both DP and MDP techniques, parallel to the incisal edges of the central incisors while the mouth was open and at approximately 1 mm from the incisal edge of the right central incisor [9, 23, 27]. Therefore, two additional techniques (DP-WB and MDP-WB) were investigated. Cross-polarization filters (CP) were used to avoid light reflections and ensure the standardization of illumination for the images. Before photo shooting, filters were used in combination with the DP and MDP techniques, and the shootings were performed with the same procedures mentioned above [27, 28]. Therefore, two more techniques (DP-CP and MDP-CP) were investigated. In addition, both the cross-polarization and white balance techniques were used together with both the DP and MDP techniques. Thus, two more photography methods, DP-CP-WB and MDP-CP-WB, were investigated in this study.

### Quantitative color assessments

For the RP process, hybrid data were recorded in.JPEG format in the device’s file inventory for each participant. The files were transferred to a computer and processed for digital color measurements using the dedicated RP software. In the software, the color change section in the ‘analysis tab’ was opened, and the green cursor on the tooth is positioned at the approximately middle point of the tooth crown by the eye and the specific L*, a*, and b* color parameters were measured and recorded per participant (Fig. 6**)**. This measurement process on the device screen for each of the captured image was repeated three times (L_1_, L_2_, L_3_, a_1_, a_2_, a_3_, b_1_, b_2_, and b_3_) by the same observer to evaluate the intraobserver correlation and minimize measurement bias.

**Fig. 7:**
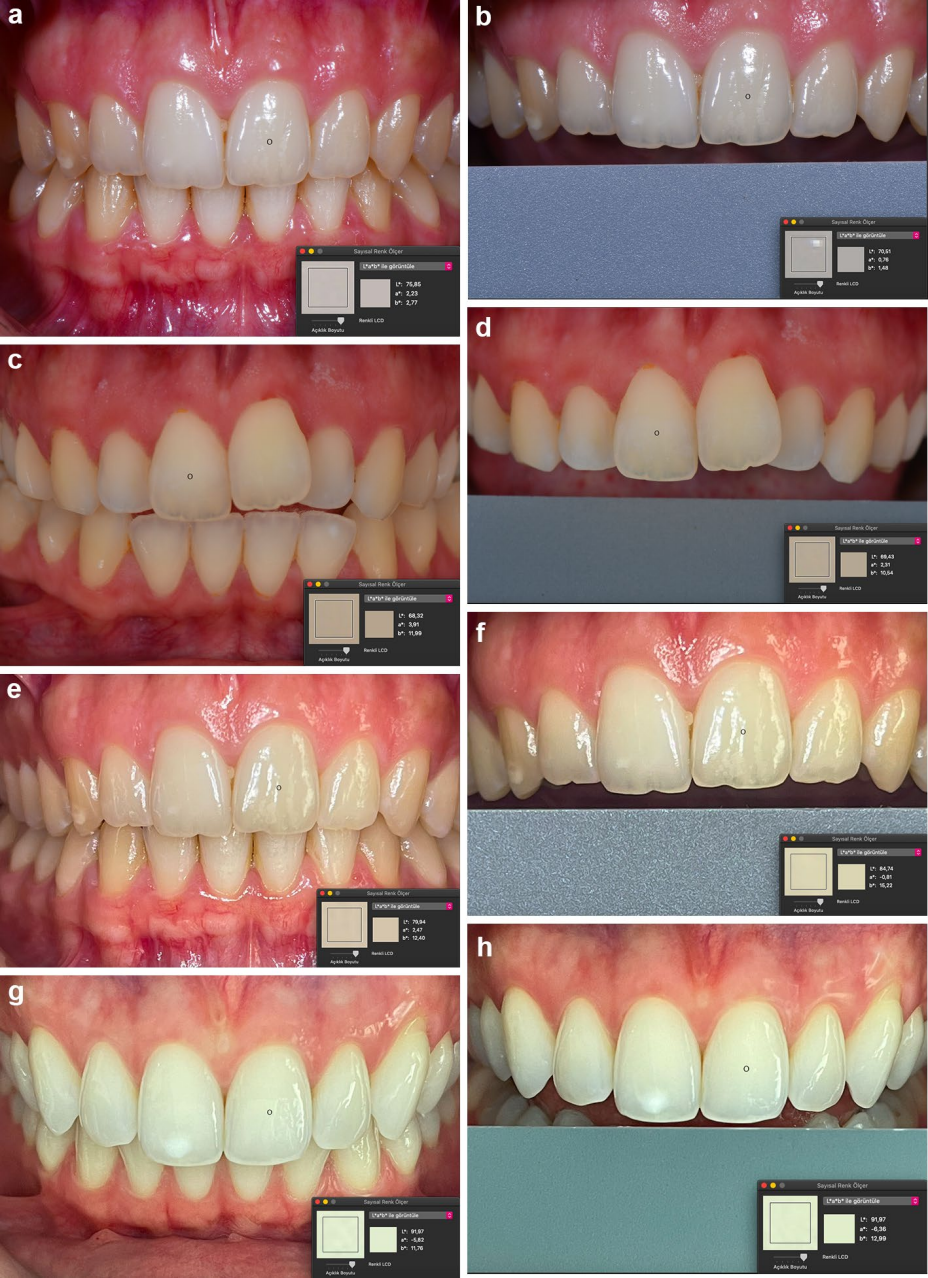
**a** DP image. **b** DP/WB image. **c** DP/CP image. **d** DP/CP/WB image. **e** MDP image. **f** MDP/WB image. **g** MDP/CP image. **h** MDP/CP/WB image

Regarding the photography techniques, the images were saved in.JPEG format for each participant according to the dehydration and dehydration period. Quantitative color measurements were performed in a computer software program (Digital Color Meter, Version 1.8.1, Macintosh AC, USA) (Figs. 7a–h) [3, 29]. L*, a*, and b* color parameters were generated for each captured image from approximately the mid-point of the middle third and three measurements (L_1_, L_2_, L_3_, a_1_, a_2_, a_3_, b_1_, b_2_, and b_3_) were done by the observer in every collected image to evaluate the intraobserver correlation. CIEDE 2000 color differences (∆E_00_*) were calculated using the generated L*, a*, and b* parameters of RP and dental photography techniques in dehydration and rehydration periods through the formula:

$$\Delta E00\, = \,\left[ {\left( {\Delta L^{\prime}/K_{L} S_{L} } \right)^{2} \, + \,\left( {\Delta C^{\prime}/K_{C} S_{C} } \right)^{2} \, + \,\left( {\Delta H^{\prime}/K_{H} S_{H} } \right)^{2} \, + \,R_{T} \left( {\Delta C^{\prime}/K_{C} S_{C} } \right)\left( {\Delta H^{\prime}/K_{H} S_{H} } \right)} \right]^{{{\raise0.7ex\hbox{$1$} \!\mathord{\left/ {\vphantom {1 2}}\right.\kern-0pt} \!\lower0.7ex\hbox{$2$}}}}$$

Data were analyzed with IBM SPSS V23. Compliance with normal distribution was analyzed using the Shapiro–Wilk test and Kolmogorov–Smirnov test. For the dehydration periods, only the MDP/WB data were normally distributed, while the rest were not normally distributed. For the rehydration periods, the MDP/CP and the MDP/WB/CP data were normally distributed, while the rest were not. Repeated analysis of variance was used to compare normally distributed delta E values three or more times, and multiple comparisons were analyzed with the Bonferroni test. Friedman’s test was used to compare data that were not normally distributed three or more times, and Dunn’s test analyzed multiple comparisons. Dependent Two-sample t-test was used to compare the ∆E_00_ values of RP and other techniques that conformed to normal distribution. Wilcoxon test was used to compare the ∆E values of RP and other techniques that did not conform to normal distribution. The intraclass correlation coefficient was used to examine the agreement between RP and other color assessment techniques and between the repetitive measurements of the observer. The significance level was taken as *p* < 0.05.

## Results

The evaluation of the agreement across the three time points for L*, a*, and b* measurements by the researcher revealed a very high level of intraobserver consistency for each color parameter, with statistical significance (*p* < 0.001 for each) (Table 3).

**Table 3:** Examination of intraobserver agreement in terms of L*, a*, and b* parameters

	ICC (%95 CI)	*p*
L*	0.99 (0.99–0.99)	** < .001**
a*	0.94 (0.93–0.94)	** < .001**
b*	0.99 (0.99–0.99)	** < .001**

ICC (95%CI): In-class correlation coefficient (95% confidence interval)

**Fig. 8:**
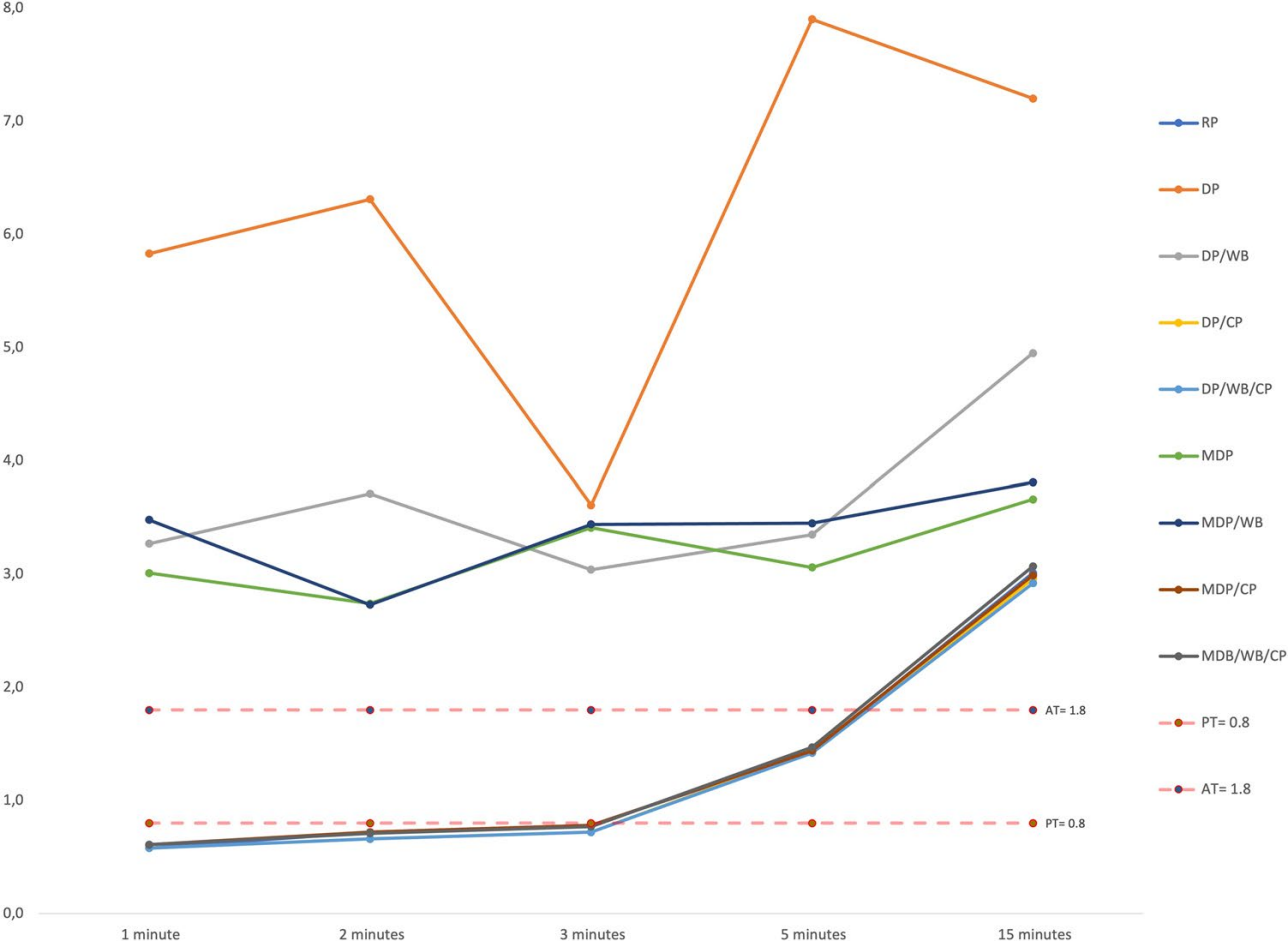
The line chart presenting the color changes per the assessment technique during dehydration

Regarding the RP scores for dehydration (Fig. 8), ∆E_00_ in the 2nd min was significantly higher than in the 1st min (*p* < 0.001), while there was no difference in the 2nd and 3rd min (*p* ≥ 0.05). The ∆E_00_ at the 5th min was significantly higher than the 3rd min (*p* < 0.001) and ∆E_00_ at the 15th min was the highest among all. The ∆E_00_ in the first 3 min was imperceptible (< 0.8); however, it was perceptible (≥ 0.8) in the 5th min, though still acceptable (< 1.8). It was not acceptable at the 15th min (≥ 1.8) (Table 4). For the DP and DP/WB scores, no significant difference was found for ∆E_00_ values at 1, 2, 3, 5, and 15 min (*p* = 0.249 and *p* = 0.134, respectively) and all readings were unacceptable. For MDP and MDP/WB scores, a nonsignificant difference was found for ∆E_00_ values at 1, 2, 3, 5, and 15 min (*p* = 0.598 and *p* = 0.083, respectively) and all readings were unacceptable. For the DP/CP and MDP/CP scores, ∆E_00_ significantly and gradually increased from the 2nd min to the 15th min for both (*p* < 0.001). The ∆E_00_ values in the first 3 min for both techniques were below the PT, however, above in the 5th min. The readings for both techniques were above the AT at the 15th min. For the DP/WB/CP and MDP/WB/CP scores, there was no difference for ∆E_00_ between the 1st and 2nd min (*p* ≥ 0.05), while the 3rd min was significantly higher than the 2nd min (*p* < 0.001 for both). ∆E_00_ at the 5th min was significantly higher than the 3rd min (*p* < 0.001 for both). The ∆E_00_ values for both techniques in the first 3 min were below the PT, however, it was above in the 5th min. The readings for both techniques were above the AT at the 15th min.

**Table 4:** Comparison of ∆E* values for dehydration periods for each technique

	1 min		2 min		3 min	5 min		15 min		Test stat		*p*
RP	0.58 (0.37–0.77)^d^		0.72 (0.57–0.87)^c^		0.78( 0.59–1.08)^c^	1.43 (1.11–2.6)^b^		3.01 (0.51–2.96)^a^		85		< .001**
DP	5.83 (0.44–28.39)		6.31 (1.26–27.91)		3.61 (0.44–23.93)	7.9 (1.19–20.54)		7.2 (1.23–22.52)		4.12		.249**
DP/WB	3.27 (0.11–23.8)		3.71 (0.82–22.65)		3.04 (0.45–22.36)	3.35 (0.66–15.58)		4.95 (0.89. –23.88)		8.68		.134**
DP/CP	0.58 (0.38–0.72)^e^		0.66 (0.5–0.88)^d^		0.72 (0.57–1.01)^c^	1.43 (0.78–2.46)^b^		2.96 (0.73–3.07)^a^		88.84		< .001**
DP/WB/CP	0.58 (0.39–0.7)^d^		0.66 (0.48–0.9)^d^		0.72 (0.58–0.98)^c^	1.42 (0.77–2.47)^b^		2.92 (0.74–2.91)^a^		87.76		< .001**
MDP	3.01 (0.31–64.86)		2.74 (0.89–66.28)		3.41 (1.29–64.94)	3.06 (1.5–65.03)		3.66 (1.8–69.21)		1.88		.598**
MDP/WB	3.48 ± 1.94		2.73 ± 1.14		3.44 ± 1.52	3.45 ± 1.44		3.81 ± 1.73		2.301		.083*
MDP/CP	0.61 (0.4–0.75)^e^		0.72 (0.54–0.9)^d^		0.78 (0.61–1.15)^c^	1.44 (0.88–1.92)^b^		2.99 (0.93–2.02)^a^		90		< .001**
MDP/WB/CP	0.61 (0.36–0.76)^d^		0.71 (0.53–0.93)^d^		0.77 (0.6–1.16)^c^	1.47 (0.86–1.94)^b^		3.07 (1.06. –2.11)^a^		87.76		< .001**

*RP* RayPlicker, *DP* digital photography, *CP* cross-polarization filter, *WB* white balanced calibration card, *MDP* mobile dental photography device

*repeated variance analysis

**Friedman test

^a^^−^^e^: No difference between durations with the same letter

**Fig. 9:**
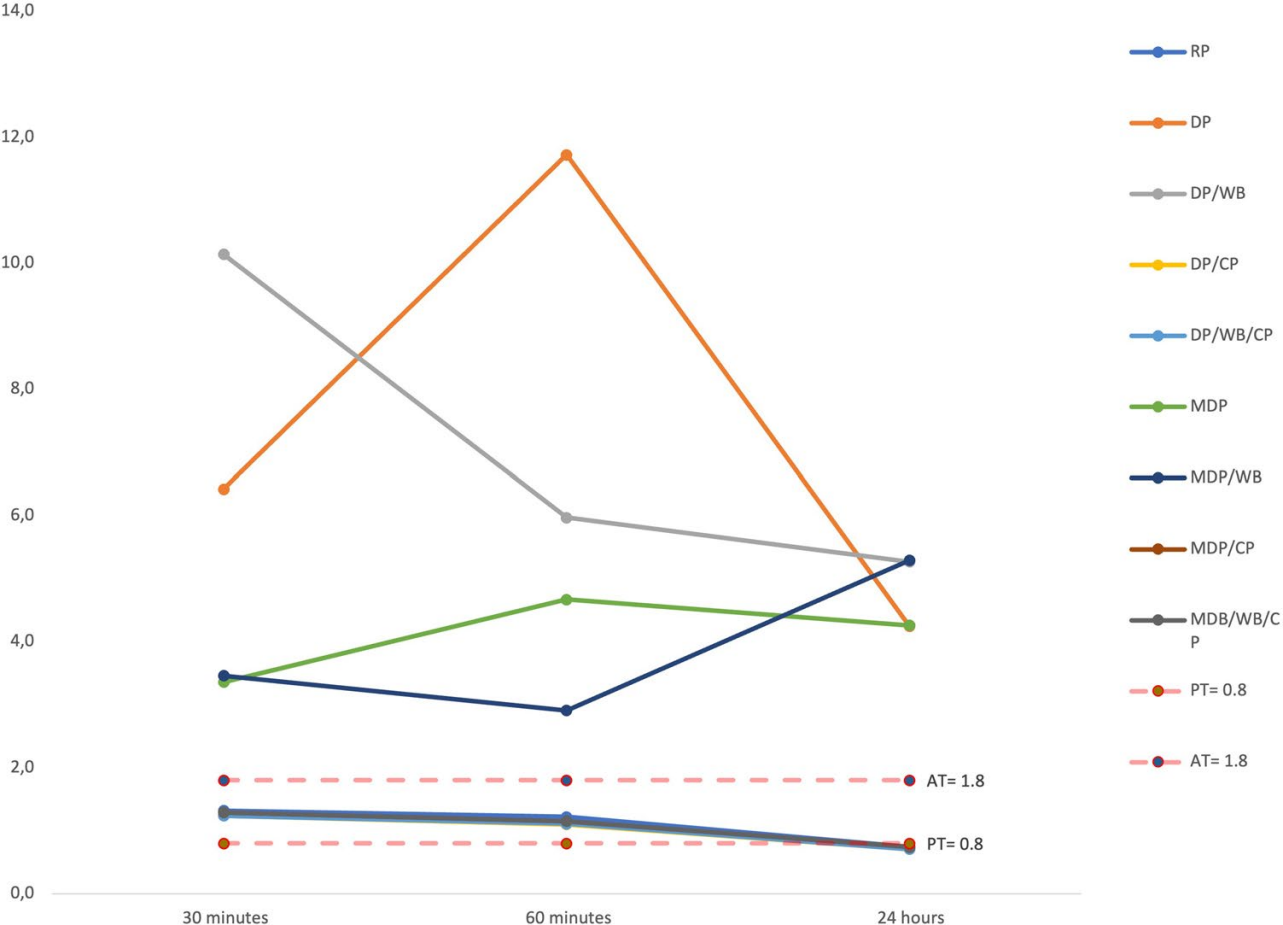
The line chart presenting the color changes per the assessment technique during rehydration

Regarding the RP, DP/CP, DP/WB/CP, MDP/CP, and MDP/WB/CP scores for rehydration (Fig. 9), ∆E_00_ at 60 min was significantly lower than the 30 min (*p* < 0.001 for all). ∆E_00_ at 24 h was significantly lower than 60 min (*p* < 0.001 for all). ∆E_00_ values were perceptible (≥ 0.8) at 30 min and 60 min, while it was imperceptible (70% of the teeth) were < 0.8) at 24 h (Table 5). For the DP and DP/WB scores for rehydration, no significant difference was found in ∆E_00_ for 30 min, 60 min, and 24 h (*p* = 0.177 and *p* = 0.531 respectively). The ∆E_00_ at 30 min, 60 min, and 24 h were unacceptable. For the MDP scores, the ∆E_00_ at 60 min was significantly higher than 30 min (*p* = 0.001). There was no difference in ∆E_00_ between 24 h and 60 min (*p* ≥ 0.05). The ∆E_00_ at 30 min, 60 min, and 24 h were unacceptable. For the MDP/WB scores, there was no difference in ∆E_00_ between 60 and 30 min (*p* ≥ 0.05). The ∆E_00_ at 24 h was significantly higher than 60 min (*p* = 0.002). The ∆E_00_ at 30 min, 60 min, and 24 h were unacceptable.

**Table 5:** Comparison of ∆E_00_ values for rehydration periods for each technique

	30 min		60 min		24 h		Test stat		*p*
RP	1.32 (1.02–2.23)^c^		1.22 (0.93–2.21)^b^		0.74 (0.5–0.99)^a^		56.267		< .001**
DP	6.42 (0.89–16.46)		11.72 (0.67–19.48)		4.25 (0.64–29.51)		3.467		.177**
DP/WB	10.14 (1.31–36.47)		5.97 (0.48–28.89)		5.27 (0.76–24.9)		1.267		.531**
DP/CP	1.24 (1.05–2.17)^c^		1.1 (0.83–1.92)^b^		0.71 (0.44–0.93)^a^		60		< .001**
DP/WB/CP	1.24 (1.03–2.18)^c^		1.11 (0.82–1.92)^b^		0.71 (0.41–0.93)^a^		60		< .001**
MDP	3.36 (0.8–63.29)^b^		4.67 (1.26–66.04)^a^		4.26 (0–65.38)^a^		14.712		.001**
MDP/WB	3.46 (1.35–12.89)^b^		2.91 (1.13–10.29)^b^		5.29 (0.45–15.6)^a^		12.067		.002**
MDP/CP	1.29 ± 0.19^c^		1.15 ± 0.2^b^		0.74 ± 0.11^a^		174.077		< .001*
MDP/WB/CP	1.29 ± 0.19^c^		1.15 ± 0.2^b^		0.74 ± 0.11^a^		172.555		< .001*

*DP* digital photography, *CP* cross-polarization filter, *WB* white balanced calibration card, *MDP* mobile dental photography device

*repeated variance analysis

**Friedman test

a-c: No difference between durations with the same letter

Very high agreements were observed for the assessments by DP/CP, DP/WB/CP, MDP/CP, and MDP/WB/CP techniques, and the RP (*p* < 0.001 for each technique) in both the dehydration and the rehydration periods (Table 6). However, no significant agreement was observed between the assessments by DP, DP/WB, MDP, and MDP/WB techniques, and the RP (*p* = 0.445, *p* = 0.437, *p* = 0.451, and *p* = 0.576, respectively), in both dehydration and rehydration periods.

**Table 6:** Examination of the agreement between RP and the other techniques

	Dehydration		Rehydration		Total	
	ICC (% 95 CI)	*p*	ICC (% 95 CI)	*p*	ICC (% 95 CI)	*p*
RP-DP	0.01 (− 0.42 – 0.31)	.475	0.03 (− 0.47 – 0.36)	.441	0.02 (− 0.29 – 0.25)	.445
RP-DP/WB	− 0.02 (− 0.46 – 0.29)	.539	0.02 (-0.49 – 0.36)	.46	0.02 (− 0.28 – 0.25)	.437
RP-DP/CP	0.99 (0.99 – 0.99)	** < .001**	0.98 (0.97 – 0.99)	** < .001**	0.99 (0.98 – 0.99)	** < .001**
RP-DP/WB/CP	0.99 (0.98 – 0.99)	** < .001**	0.98 (0.97 – 0.99)	** < .001**	0.99 (0.98 – 0.99)	** < .001**
RP-MDP	0.01 (− 0.42 – 0.31)	.472	0.02 (-0.49 – 0.36)	.461	0.02 (-0.29 – 0.25)	.451
RP-MDP/WB	0.06 (− 0.35 – 0.35)	.363	− 0.14 (− 0.74 – 0.25)	.734	− 0.03 (− 0.35 – 0.22)	.576
RP-MDP/CP	0.98 (0.96 – 0.98)	** < .001**	0.95 (0.93 – 0.97)	** < .001**	0.97 (0.96 – 0.98)	** < .001**
RP-MDB/WB/CP	0.97 (0.96 – 0.98)	** < .001**	0.95 (0.93 – 0.97)	** < .001**	0.97 (0.96 – 0.98)	** < .001**

ICC (95% CI): Intraclass correlation coefficient (95% confidence interval)

## Discussion

Dehydration may influence the true color perception of a clinician due to the temporary dental color change, and it may result in an irreversible complication such as color mismatch during the restorative treatment [18]. This study clinically investigated the quantitative color changes of maxillary central incisors and evaluated the related dental color perception due to dehydration at short-term periods and the reverse effect of rehydration up to 24 h, using different dental photography techniques and a spectrophotometer. According to the results of the present study, the clinical perception of tooth color was affected by both the level of tooth dehydration and rehydration. Therefore, the first and second null hypotheses of the study were rejected. Some of the dental photography techniques presented similar results to the spectrophotometer outcomes, while some presented significant differences in clinical color detection. Therefore, the third hypothesis of the study was partially accepted.

Visual color analysis may cause inconsistency in color evaluation due to the subjectiveness, and the most precise, practical, and adaptable tool for dental color measurement was considered the dental spectrophotometers [25, 30]. The clinical contact-type spectrophotometers were found repeatable and reliable in measuring dental color parameters quantitatively [9, 25, 31]. A clinical hybrid-type (with cross-polarization feature) dental spectrophotometer was used as the control in the present study. The older versions with the same working principle were previously mentioned as the clinical standard for the non-hybrid and non-polarized feature color assessment devices due to the ability to avoid ambient light and, thereby, provide high accuracy and reliability [32]. A previous version of the spectrophotometer with the same features (SpectroShade) was reported to have 96.9% reliability and 80.2% accuracy [33]. Even though the accuracy was inferior to the non-polarized spectrophotometer (VITA EasyShade), the observed reliability was superior to it. The lower rate for accuracy was probably due to the difference in the analysis area of the two devices. EasyShade device’s small tip measures an area of only 5 mm in diameter more precisely, while hybrid-type spectrophotometers can measure the entire tooth surface more realistically and provide average tooth shade, shade by region (thirds), or detailed color map [33]. Because of the variety of colors on a particular tooth surface, similar to the previous clinical studies that focused on the middle third of the maxillary central incisors, the tip of the RP device was located on the cervical- and mid-third surface including the cervical gingival area in this study [11, 14, 34, 35]. The standardization in the positioning of all measurement equipment and camera and shooting parameters was done to avoid bias [36].

The level of color change due to dehydration or rehydration was considered time-dependent [13, 37]. Alamé et al. [16] mentioned that the more the dehydration time elapses, the more the color difference compared to the baseline increases. Du et al. [34] measured the level of dehydration in vitro at 2 h and 4 h using a colorimeter and observed a significant color change for both periods. Ahmed et al. [38] investigated the effect of dehydration in vitro with a spectrophotometer at baseline, 1 h, and 2 h and observed significant dental color changes at both periods. However, decreasing the time intervals for more precise results was recommended [17, 38]. In a clinical study, Russel et al. [11] mentioned significant color changes in maxillary central incisors after 15 min of dehydration. Burki et al. [14] observed significant color changes clinically with a spectrophotometer in maxillary central incisors after 10 min of dehydration. Ibrahim and Abou Steit [17] also used a spectrophotometer clinically to evaluate dehydration at 10 and 30 min for central incisors. They observed a significant increase in ∆E* at 10 min, supporting the results of Burki et al. [14], but no difference between 10 and 30 min. Consistent with these results, in the present study, RP ∆E_00_ values gradually and significantly increased till the 15th min (Table 4). Hatırlı et al. [13] conducted another clinical study for dehydration and rehydration of maxillary incisors using a hybrid-type spectrophotometer and evaluated dehydration for a total of 30 min. They observed significant color changes for each of the 10-min intervals supporting Burki et al.’s [14] and Ibrahim and Abou Steit’s [17] results, and the ∆E_00_ at the 10th min was above the AT (≥ 1.8). Sharmila et al. [39] conducted a clinical study with a similar methodology to Hatırlı et al. [13] comparing also younger and older patients. They presented perceptible and clinically unacceptable color changes after 10 min of dehydration like the results of Hatırlı et al. [13]. Whereas the color changes of older patients were clinically acceptable up to 30 min of dehydration, that the color brightening was less pronounced for the older patients. The RP color change levels after 15 min of dehydration in the present study were consistent with these previous findings and were above the AT, supporting the results of Hatırlı et al. [13]. However, due to the inclusion criteria of this study (participants at the age of 20–40), no statistical data were obtained for the differences between young and old patients.

All the previous study results mentioned above reported the long-term effect of dehydration on dental color. However, the dehydration effect on dental color starts at the earliest minutes of the dental appointment, which means the color should be selected before any restorative procedure [1, 2, 4, 14]. Moreover, there is limited clinical evidence in the literature regarding shorter term intervals to evaluate dehydration and longer term intervals for rehydration [12, 15, 17, 38]. Accordingly, this study investigated the short-term intervals to evaluate the dehydration clinically and the influence on the tooth color. Ruiz-López et al. [15] conducted a clinical study with short-term dehydration intervals of 2, 4, 6, and 8 min and evaluated the color changes of maxillary incisors due to dehydration using a spectroradiometer. They presented ∆E_00_* values higher than PT (≥ 0.8) for 50% of the teeth after 2 min of dehydration and 95.8% after 10 min. Their results were beyond the AT after 6 min of dehydration, consistent with the control method RP results of this study. Buldur et al. [18] evaluated the effect of dehydration and rehydration with short-term intervals (1, 2, 3, 5, 7, 10, and 15 min for each) on maxillary immature permanent central incisors using a spectrophotometer. They observed 50% surpassing the AT at the 5th min of dehydration for the teeth with completed root development. Suliman et al. [12] investigated short-term dehydration intervals of 1, 2, 3, 5, 7, 10, and 15 min clinically for maxillary incisors using a spectrophotometer and observed significant color changes within the 1st min of dehydration, which were 87% beyond the PT (0.8) and 72% beyond the AT (1.8). Inconsistent with these results, in the RP results of the present study, ∆E_00_* was beyond the PT for only 23.4% of the teeth after 3 min of dehydration but 100% at 5 min (Table 4). Therefore, a fast change in color perception was observed due to the dehydration between the 3rd and the 5th min. At the 5th min, only 16.7% of the teeth were beyond the AT, and thus, most of the teeth’s color was clinically acceptable, inconsistent with the results of Buldur et al. [18] and Suliman et al. [12]. Surpassing the AT for 100% was observed at the 15th min but not before, according to the RP outcomes. The inconsistency in the observed intervals with the previous studies for perceptibility and acceptability might be due to the differences in the standardization of the clinical setups or the type and specification of the spectrophotometer used. Especially, the hybrid spectrophotometer (RP) device used in this study was particularly produced for the clinical color assessment by combining spectrophotometric analysis with cross-polarization photography. More standardized clinical assessments could be achieved using the features of auto-perceiving of the tooth crown and related cervical gingiva and guided angulation during the shooting. On the other hand, color matching is a crucial step for restorative success, and dental color selection mistakes are irreversible during restorative procedures. Therefore, it might be wiser to consider the perceptibility clinically more important than the acceptability, which is affected in the longer term. Accordingly, the RP results of the present study revealed that it might be better to select the dental color within the very first 3 min of the dental appointment to avoid the color change due to the dehydration effect.

Following dehydration, the clinical time needed for complete rehydration is also very critical for the clinical workflow and success, and this study also clinically investigated the level of color reversal after the dehydration. Buldur et al. [18] and Suliman et al. [12] considered more than 15 min of rehydration was required to regain the original color after 15 min of dehydration. However, in these studies, the rehydration period was limited only to 15 min like most of the previous clinical studies. Inconsistent with them, Russel et al. [11] mentioned that the original color was regained after 30 min of rehydration and Burki et al. [14] supported their result. The RP results of the present study are opposed to these previous findings because 100% of the teeth were below the AT but still beyond the PT at the 30th min of rehydration. Even after the 60th min of rehydration, although ∆E_00_ significantly decreased, supporting the results of Ibrahim and Abou Steit [17], the situation regarding PT and AT was completely the same as mentioned above. However, after at least 24 h of rehydration, ∆E_00_ values according to the RP were below the PT, and 70% of the teeth regained their original color (Table 5). It can be interpreted that, even after 24 h of rehydration, there is still a probability that the original tooth color cannot be restored. This result is also consistent with the results of Hatırlı et al. [13], reporting that the color returned to the original level at least after 24 h of rehydration. 90% of the teeth were below PT at the 24th h in their results, while it was 70% in the present study, which can be considered more critical for clinical practice. Moreover, the recent results of Sharmila et al. [39] were completely consistent with the results of this study, in which they reported the ∆E* was below the PT only after 48 h of rehydration.

Digital dental photography has been used to assess the clinical assessment of dental color besides the spectrophotometers [7, 40–42]. Moazam et al. [41] clinically compared a spectrophotometer (VITA EasyShade) and DP for evaluating the color of maxillary incisors and considered digital photography a highly reliable clinical assessment method. Although this is an important outcome, they did not use the CP filters for the DP technique. Yilmaz et al. [28] compared a spectroradiometer, DP (with ring flash), and CP photography in vitro and used WB calibration for all the photographs. They observed similar outcomes for the spectroradiometer and CP photography, especially for the high-value shades. This result might be due to the ability of the CP filter to avoid unwanted light reflections on the surface, providing an advantage for the teeth with high value and gloss clinically. Korkut et al. [43] compared a spectrophotometer (VITA EasyShade V) and CP photography for the color assessment of composite samples and observed a very high positive correlation between the two techniques. He et al. [32] clinically compared a clinical hybrid-type spectrophotometer (ShadepilotTM, DeguDent, Germany), DP (with twin-flash), and CP photography in tooth color assessment and mentioned that the CP photography presented a high agreement with the spectrophotometer outcomes as control. The spectrophotometer they used was an older version of the one used in the present study. In this study, supporting He et al. [32] and Yilmaz et al. [28], all the photography techniques, including CP filters, presented very high agreements with the spectrophotometric control method RP (Table 6). Therefore, the DP/CP technique might be a clinical replacement for the spectrophotometric color analysis. The results revealed that the use of the CP filters led to more standardized images and, thereby, color assessments for monitoring both dehydration and rehydration clinically. Evaluating the DP and MDP techniques, only the assessments through the cross-polarized images were like the assessments with the RP results (Tables 4 and 5). Hein et al. [9] introduced the eLAB system, focusing on standardizing digital camera images using a specific gray card to calibrate the WB, and considered it a promising method for quantitatively measuring dental color in dental practice. Supporting their results, Swarowsky et al. [42] and Yung et al. [43] considered WB calibration to improve the determination of color differences through the photographs. However, in the present study, WB calibration individually did not improve the determination of color changes but improved when used together with the CP filters. The results obtained solely from the CP filters without the WB calibration also presented a high agreement with the RP outcomes. Thus, the DP/WB/CP technique may provide another clinical option for the spectrophotometric color analysis. He et al. [32] mentioned that CP photography can remove specular highlights and thereby, aid in the observation of subtle color changes that may not be evident in regular flash photography. The quantitative assessment through the collected images might be influenced by the glossy tooth surfaces even when calibrating the white balance [43]. This might be the reason for the lower effectiveness of WB calibration, compared to the use of CP filters in this study. The previous clinical results of Sampaio et al. [7] and Korkut et al. [43] completely agreed with these findings by considering the WB calibration not necessary when using CP filter photography. They also reported less accurate photography results with the smartphone photography and ring flash photography compared to the CP photography, consistent with our results (Table 6). Whereas better results for both the MDP and DP techniques were obtained in this study when they were used with the CP filters. This inconsistency was probably due to the absence of external illumination like the MDP device and the CP filters for the smartphone photography technique in Sampaio et al.’s study [7]. Supporting the results of Yung et al. [43], the results of this study can be interpreted that smartphone photography (MDP/CP or MDP/WB/CP technique) can be used effectively for clinical color assessment and as an alternative to the spectrophotometers when it is used together with an extraoral illumination like an MDP device and the CP filters. Jorquera et al. [23] supported this by considering MDP (with the CP filter) and CP photography methods comparable and reliable for dental shade selection in a clinical study. Consistent with their statement, WB cards, and CP filters can be thereby beneficial tools to standardize dental colors in terms of communication with dental technicians [23]. On the contrary, two recent clinical studies considered CP photography alone was not beneficial for color assessment, inconsistent with our results [31, 43]. However, Yung et al. [43] used no spectrophotometric control method in their study and captured all the images under fluorescence lightning, but not the daylight at 5500 Kº. They also used the old CIELab76 formula to calculate the color changes which was not as definitive as the CEIDE2000. Moreover, they captured all the images under fluorescence lightning, but not the daylight at 5500 Kº. Saygılı et al. [31] used a non-polarized spectrophotometer device (EasyShade) as a control which can analyze only a limited surface area of 5 mm and be affected by the ambient light that could have influenced the obtained color data. CP photography might be affected by the experience level of the observer, depending on their reports. In addition, both studies investigated only the young participants and did not mention the dehydration level during the photo shooting procedures, which might also have affected their results and caused the inconsistency. These might be the possible reasons for the inconsistency of these studies with our results.

In the present study, there were no major differences in effectiveness between the investigated dental photography techniques that involved the CP filters, and those were considered clinically promising, whereas the ones not containing the CP filters were not considered clinically convenient under the limitations of this study. However, there might be some limitations. It might be more accurate to assess the dental color changes by also using other clinical spectrophotometric analysis devices and to check the agreement level with the current techniques. Moreover, other brands of cross-polarization filters, white balance calibration cards, and dental photography equipment should be investigated, which may influence the outcomes. In addition, other computer-based software programs can be used for the quantitative analyses, and the influence on the outcomes should be evaluated.

## Conclusions

Within the limits of this study, it was concluded that

1. Selecting the dental color within the first 3 min of the appointment is safer to avoid perceptible dehydration effects on color.
2. The original color may not be fully restored, and as a result, the color might still look brighter even after 24 h of rehydration following 15 min of dehydration.
3. Cross-polarization filters are essential tools that play the most important role in standardizing the images for clinical tooth color assessment, whereas white balance calibration individually did not improve the determination of color changes. Smartphones can be used for clinical assessment of dental color effectively only if used together with extraoral illumination such as MDP devices and CP filters.

## Data Availability

The data that support the findings of this study are available on request from the corresponding author.
